# Biotransformation of ginsenosides Rb_1_, Rg_3 _and Rh_2 _in rat gastrointestinal tracts

**DOI:** 10.1186/1749-8546-5-19

**Published:** 2010-05-26

**Authors:** Tianxiu Qian, Zongwei Cai

**Affiliations:** 1 Department of Chemistry, Hong Kong Baptist University, Kowloon Tong, Kowloon, Hong Kong SAR, China; 2 Institute of Medicinal Plant Development, Chinese Academy of Medical Sciences and Peking Union Medical College, Beijing 100193, China

## Abstract

**Background:**

Ginsenosides such as Rb_1_, Rg_3 _and Rh_2 _are major bioactive components of *Panax ginseng*. This *in vivo *study investigates the metabolic pathways of ginsenosides Rb_1_, Rg_3 _and Rh_2 _orally administered to rats.

**Methods:**

High performance liquid chromatography-mass spectrometry (LC-MS) and tandem mass spectrometry (MS-MS) techniques, particularly liquid chromatography electrospray ionization mass spectrometry (LC-ESI-MS), were used to identify the metabolites.

**Results:**

Six metabolites of Rb_1_, six metabolites of Rg_3 _and three metabolites of Rh_2 _were detected in the feces samples of the rats. Rh_2 _was a metabolite of Rb_1 _and Rg_3_, whereas Rg_3 _was a metabolite of Rb_1_. Some metabolites such as protopanaxadiol and monooxygenated protopanaxadiol are metabolites of all three ginsenosides.

**Conclusion:**

Oxygenation and deglycosylation are two major metabolic pathways of the ginsenosides in rat gastrointestinal tracts.

## Background

*Panax ginseng *(*Renshen*) is used in Chinese medicines to treat various conditions such as debility, ageing, stress, diabetes, insomnia and sexual inadequacy [1-3]. The major bioactive components of *P. ginseng *are O-glycosides of the triterpen dammarane saponins known as ginsenosides [4,5] which exhibit properties such as anti-inflammation and anti-tumor [6-8]. Over 80 ginsenosides have been isolated from *P. ginseng *[9]. Rb_1_, Rg_3 _and Rh_2 _are three major ginsenosides with various bioactivities.

Rb_1_, which is the most abundant (0.22-0.62%) among all ginsenosides [5], protects against free radical damage, maintains normal cholesterol and blood pressure [10] and inhibits the induction phase of long-term potentiation by high frequency stimulation in the dentate gyrus of the brain [11]. Rb_1 _also rescues hippocampal neurons from lethal ischemic damage [12] and delays neuronal death from transient forebrain ischemia *in vitro *[13]. Rg_3 _is used as the major active component in an anti-tumor and anti-cancer drug in China [14]. The cytotoxicity of ginsenoside Rg_3 _against tumor cells increases when Rg_3 _is metabolized into Rh_2 _or protopanaxadiol [15]. The metabolic transformation of Rg_3 _into protopanaxadiol also increases the activity against *Helicobacter pylori*. Recently, *in vitro *biotransformation of ginsenosides was reported. The metabolites were identified by high-resolution tandem mass spectrometry. Degradation and bioconversion routes of the different ginsenosides at acidic (gastric) conditions and in the presence of intestinal microbiota were elaborated [16].

High performance liquid chromatography (HPLC) is a powerful chemical analysis technology that allows complex mixtures to be transformed into separated components. Mass spectrometry (MS) has progressed extremely rapidly during the last decade; especially in production, separation and ejection of ions, data acquisition and data reduction. Compared to other detectors, the advantages of the mass spectrometer are that in many cases it can provide absolute identification, not only structural information from the molecule under investigation but the molecular weight of the analyte.

Due to the specificity and sensitivity of LC-MS, especially in combination with MS-MS, it is powerful in identification of drug metabolites. Common biotransformation, *e.g*., oxidative reactions (hydroxylation), conjugation reactions to produces sulphates, glucuronides, glutathiones or other conjugates, hydrolysis of esters and amides, and reduction reactions, can be evaluated from just the knowledge of the molecular mass of the metabolites. Combination of the molecular-mass and possible biotransformation products, predicted by computer-aided molecular modeling approaches, enables the confirmation of metabolic pathways. Further confirmation and/or structure elucidation of metabolites is possible using MS-MS methods [17]. The identification of the metabolites of antihistamine compounds is feasible by using thermospray LC-MS and LC-MS-MS [18,19]. The present study aims to investigate the biotransformation of ginsenosides Rb_1_, Rg_3 _and Rh_2 _orally administered to rats by using LC-MS and MS-MS.

## Methods

### Chemicals

Ginsenosides Rb_1_, Rg_3 _and Rh_2 _(purity >99%) were provided by the Chinese Medicine Laboratory, Changchun Institute of Applied Chemistry, Chinese Academy of Sciences, China. HPLC-grade methanol was purchased from Acros Organics (USA). A Mili-Q Ultra-pure water system (Millipore, USA) was used to provide water for all the experiments. Other chemicals (analytical grade) were purchased from Sigma (USA).

### Administration of ginsenosides

Water soluble Rb_1_, Rg_3 _and Rh_2 _were administered to three groups (*n *= 3 in each group) of male Sprague Dawley rats (body weight 200-220 g; age 6-7 weeks) respectively at a dose of 100 mg/kg body weight with 2 ml dosing solution. The protocols of the animal study were fully complied with the University policy on the care and use of animals and with related codes of practice. The animal experiments were conducted with the licenses granted by Hong Kong Hygiene and Health Department. Rat feces samples were collected at such intervals: 0 to 120 hours for Rb_1 _(half-life 16.7 hours), 0 to 24 hours for Rg_3 _(half-life 18.5 minutes) and 0 to 48 hours for Rh_2 _(half-life 16 minutes)[20-22].

### Feces sample preparation

Each feces sample of each rat was suspended in 150 ml of water and then extracted with n-butanol (100 ml × 3). The extract was dried and the residue was dissolved in 1 ml of methanol. After centrifugation at 12000 rpm for 20 minutes (Eppendorf Centrifuge 5415R, Hamburg, Germany), 2 μl of the supernatant was analyzed with LC-Ms and LC-MS-MS for the identification of the ginsenosides and their metabolites. The blank feces (baseline) were collected from the same Sprague Dawley rat prior to the administration of ginsenosides, prepared and analyzed with the same method as the experimental groups.

### LC-ESI-MS analysis

HPLC separation was performed with a LC system coupled with an auto-sampler and a micro mode pump (HP1100, Agilent Technologies, USA). A reversed-phase column (Waters, Xterra MS-C8, 2.1 × 100 mm, 3.5 μm) was used to separate the ginsenosides and their metabolites. The auto-sampler was set at 10°C. Mobile phase consisted of two eluents: water (A) and methanol (B). Gradient elution was 40% B in 0-4 minutes, 40-90% B in 4-5 minutes, 90% B in 5-35 minutes, 90-40% B in 35-36 minutes and 40% B in 36-42 minutes at a flow rate of 100 μl/min. Effluent from the LC column was diverted to waste for the first 12 minutes following the injection, and then diverted to the MS ion source.

MS experiments were performed on a quadruple-time of flight (Q-TOF) tandem mass spectrometer API Q-STAR Pulsar I (Applied Biosystems, USA). Negative or positive ion mode in electrospray ionization (ESI) was used to analyze ginsenosides and their metabolites in rat feces samples. The following parameters of the turbo-ionspray for positive ion mode were used: ionspray voltage 5500 V, declustering potential 1 (DP1) 90 V, focusing potential (FP) 265 V and declustering potential 2 (DP2) 10 V, collision energy (CE) 55 eV for MS-MS analysis. For negative ion mode, the parameters were: ionspray voltage -4200 V, declustering potential 1 (DP1) -90 V, focusing potential (FP) -265 V and declustering potential 2 (DP2) 10 V, collision energy (CE) -60 eV for MS-MS analysis. For both positive and negative ion mode, the ion source gas 1 (GS1), gas 2 (GS2), curtain gas (CUR) and collision gas (CAD) were 20, 15, 25 and 3, respectively. The temperature of GS2 was set at 400°C.

## Results and Discussion

### Metabolites of Rb_1 _in rat feces

**Figure 1:**
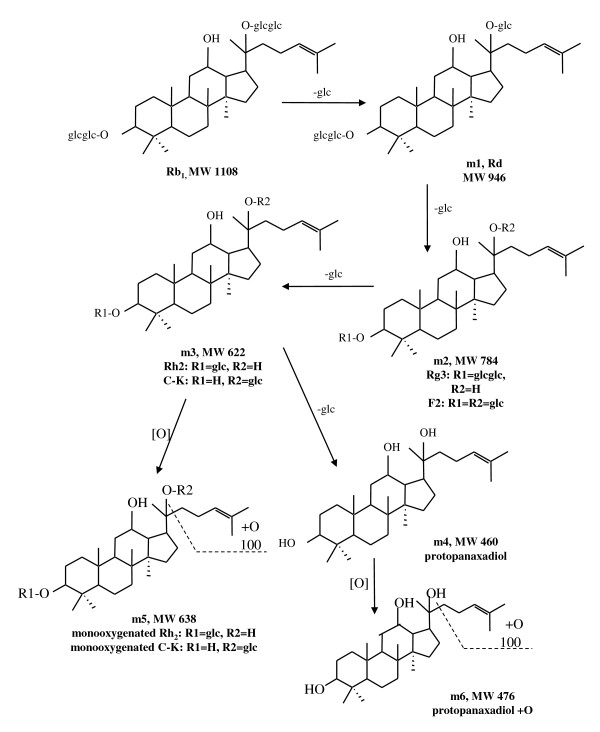
**Deglycosylated and oxygenated metabolic pathways of Rb_1 _orally administered to rat*s***.

**Figure 2:**
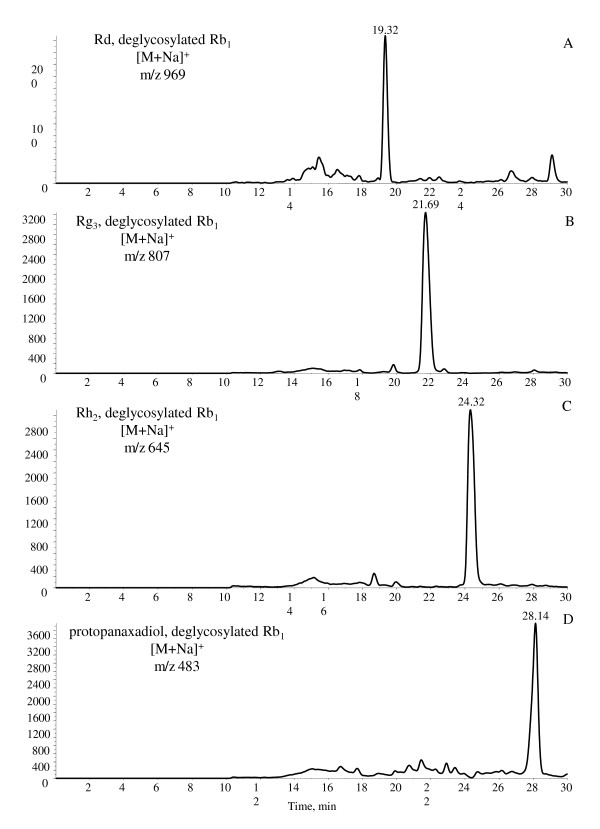
**MS spectra of Rb_1 _orally administered to rats**. (A) Rd and its deglycosylated metabolites, *m/z *969; (B) Rg_3_, *m/z *807; (C) Rh_2_, *m/z *645; (D) protopanaxadiol, *m/z *483.

**Figure 3:**
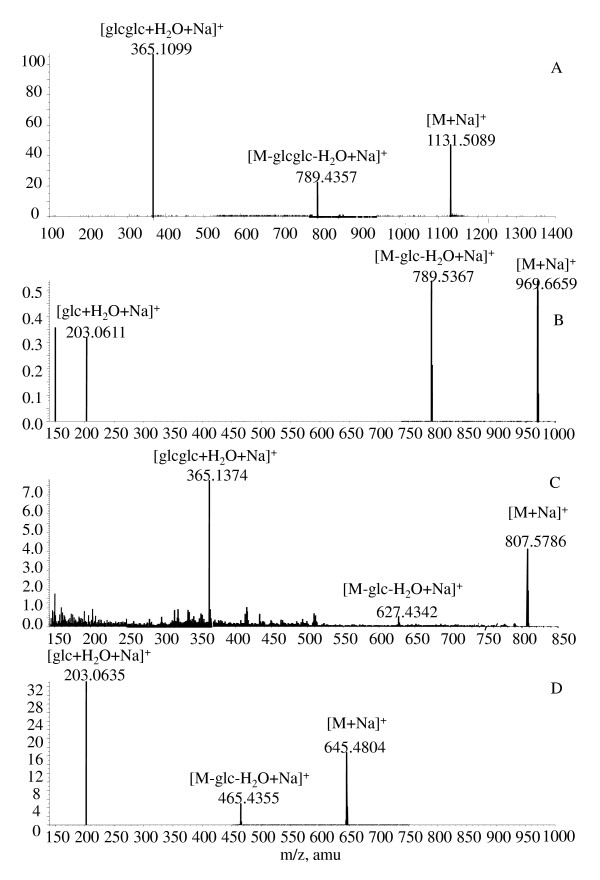
**LC-MS-MS spectra of ginsenosides**. (A) Rb_1 _and its deglycosylated metabolites; (B) Rd; (C) Rg_3_; (D) Rh_2_.

The parent Rb_1 _and direct oxygenated metabolites of Rb_1 _were not detected in the feces samples. These results suggested that Rb_1 _might have largely metabolized in the gastrointestinal tracts in rats. Six metabolites were detected in rat feces samples collected 0-120 hours after Rb_1 _was orally administered (Figure 1). The metabolites were detected from the LC-MS analyses and confirmed by the results from the LC-MS-MS experiments in positive ESI mode [18]. A total of four deglycosylated metabolites were identified, namely Rd, Rg_3_, Rh_2 _and protopanaxadiol (Figure 2). Analysis of [M + Na]^+ ^ions (Figure 3) indicated that the metabolites shared similar MS-MS fragmentation pattern with the parent Rb_1_. The fragmentation patterns of the metabolites, produced from the [M + Na]^+ ^ions at *m/z *969, *m/z *807, and *m/z *645 respectively, were compared with that of Rb_1_. The deglycosylated metabolites of Rb_1 _showed the same fragment patterns as Rb_1_, i.e. the glucose moiety and water were lost from the molecular ion and the corresponding sodium-adduct daughter ions at *m/z *789 and *m/z *203 for Rd, *m/z *627 and *m/z *365 for Rg_3 _and *m/z *465 and *m/z *203 for Rh_2 _were produced.

The deglycosylated metabolites were also confirmed by the LC-MS analysis of authentic standards of Rd, Rg_3_, Rh_2 _and protopanaxadiol. Moreover, the LC-MS-MS analysis indicated that these deglycosylated metabolites were subsequently oxygenated in digestive tracts. Thus, deglycosylation and subsequent oxygenation are the major metabolic pathways of orally administered Rb_1 _in rats. Figure 1 illustrates the proposed metabolic pathways of Rb_1_.

### Metabolites of Rg_3 _in rat feces

**Figure 4:**
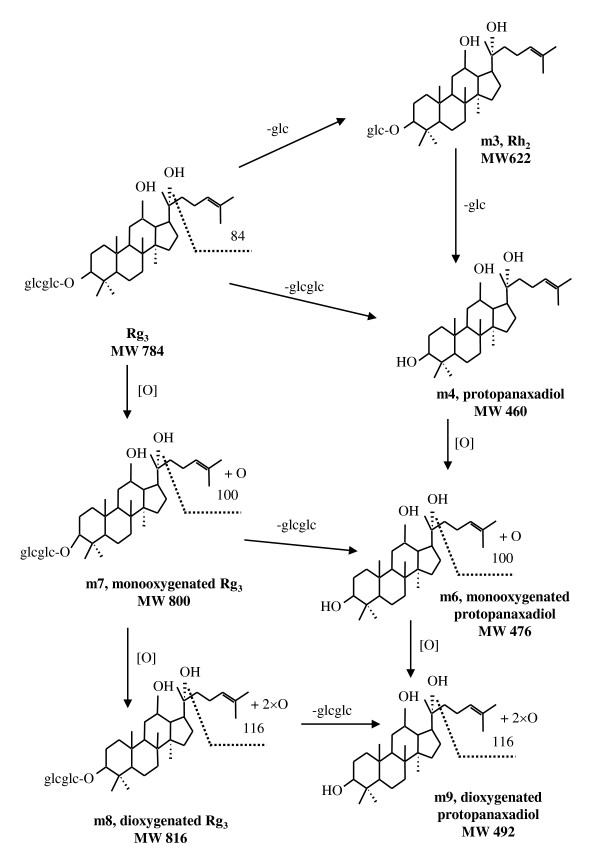
**Metabolic pathways of Rg_3 _orally administered to rats**.

Six metabolites were detected in rat feces samples collected 0-24 hours after Rg_3 _was orally administered. The same LC-MS and MS-MS method as for Rb_1 _was used to detect major deglucosylated and further oxygenated metabolites of Rg_3_. The MS-MS results were similar to those for Rb_1_. Rh_2 _and protopanaxadiol as the deglucosylated products were also confirmed by reference standards. Figure 4 summarizes the major metabolites of Rg_3 _detected in the rat feces samples and the metabolic pathway in rat gastrointestinal tracts. After the oral administration, oxygenation and deglycosylation appeared to be the major metabolic pathways of ginsenosides. Metabolites were detected for the parent Rg_3 _and its deglucosylated metabolites including the mono- and deoxygenated products of protopanaxadiol.

### Metabolites of Rh_2 _in rat feces

**Figure 5:**
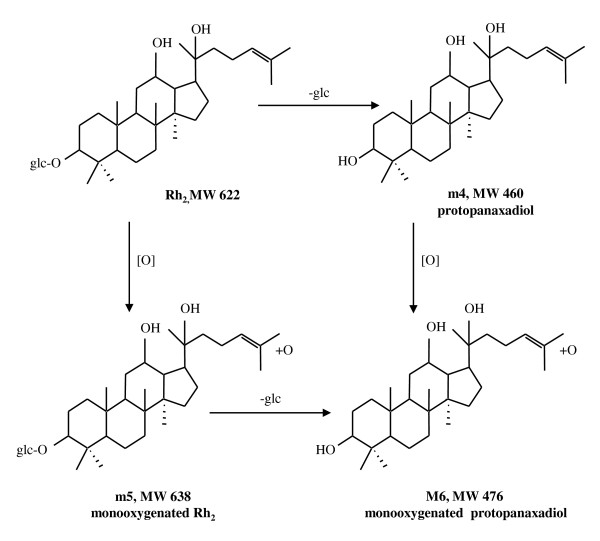
**Metabolic pathways of Rh_2 _orally administered to rats**.

Three major metabolites were detected in rat feces samples collected 0-48 hours after Rh_2 _was orally administered. The LC-MS and MS-MS method in positive ESI mode was used to detect and confirm the metabolites respectively. Oxygenated products, such as mono-oxygenated protopanaxadiol, were also identified. Deglycosylation and oxygenation were the major metabolic pathways of Rh_2_. Figure 5 illustrates the proposed metabolic pathway of Rh_2 _in rat gastrointestinal tracts.

## Conclusion

Oxygenation and deglycosylation are two major metabolic pathways of the ginsenosides in rat gastrointestinal tracts. Furthermore, Rh_2 _is a metabolite of Rb_1 _and Rg_3_, whereas Rg_3 _is a metabolite of Rb_1_. Some metabolites such as protopanaxadiol and monooxygenated protopanaxadiol are metabolites of all three ginsenosides.

## Abbreviations

HPLC: High performance liquid chromatography; LC-MS: High performance liquid chromatography coupled with mass spectrometry; MS-MS: Tandem mass spectrometry; LC-MS-MS: High performance liquid chromatography coupled with tandem mass spectrometry; ESI: Electric-spray ionization; Q-TOF: Quadruple-time of flight; DP: Declustering potential; CE: Collision energy; EP: Focusing potential; GS: source gas; CUR: Curtain gas; CAD: Collision gas; LC-ESI-MS: Liquid chromatography electrospray ionization mass spectrometry.

## Competing interests

The authors declare that they have no competing interests.

## Authors' contributions

TXQ designed the experimental study, conducted the animal and LC-MS experiments and performed the analysis. ZWC conceived the study. All authors read and approved the final manuscript.
